# Diversity and enterotype in gut bacterial community of adults in Taiwan

**DOI:** 10.1186/s12864-016-3261-6

**Published:** 2017-01-25

**Authors:** Chao Liang, Han-Chi Tseng, Hui-Mei Chen, Wei-Chi Wang, Chih-Min Chiu, Jen-Yun Chang, Kuan-Yi Lu, Shun-Long Weng, Tzu-Hao Chang, Chao-Hsiang Chang, Chen-Tsung Weng, Hwei-Ming Wang, Hsien-Da Huang

**Affiliations:** 1 Institute of Bioinformatics and Systems Biology, National Chiao Tung University, HsinChu, Taiwan; 2 Tseng Han-Chi’s General Hospital, Nantou, Taiwan; 3 Health GeneTech Corporation, Taoyuan, Taiwan; 4 Department of Biological Science and Technology, National Chiao Tung University, HsinChu, Taiwan; 5 Department of Obstetrics and Gynecology, Hsinchu Mackay Memorial Hospital, Hsinchu, Taiwan; 6 Mackay Medicine, Nursing and Management College, Taipei, Taiwan; 7 Department of Medicine, Mackay Medical College, New Taipei City, Taiwan; 8 Graduate Institute of Biomedical Informatics, Taipei Medical University, Taipei, Taiwan; 9 School of Pharmacy, College of Pharmacy, Taipei Medical University, Taipei, Taiwan; 10 Department of Biomedical Science and Environmental Biology, Kaohsiung Medical University, Kaohsiung, Taiwan

**Keywords:** Enterotype, 16S rDNA, Next-generation sequencing, Gut microbiome, Predictive model

## Abstract

**Background:**

Gastrointestinal microbiota, particularly gut microbiota, is associated with human health. The biodiversity of gut microbiota is affected by ethnicities and environmental factors such as dietary habits or medicine intake, and three enterotypes of the human gut microbiome were announced in 2011. These enterotypes are not significantly correlated with gender, age, or body weight but are influenced by long-term dietary habits. However, to date, only two enterotypes (predominantly consisting of *Bacteroides* and *Prevotella*) have shown these characteristics in previous research; the third enterotype remains ambiguous. Understanding the enterotypes can improve the knowledge of the relationship between microbiota and human health.

**Results:**

We obtained 181 human fecal samples from adults in Taiwan. Microbiota compositions were analyzed using next-generation sequencing (NGS) technology, which is a culture-independent method of constructing microbial community profiles by sequencing 16S ribosomal DNA (rDNA). In these samples, 17,675,898 sequencing reads were sequenced, and on average, 215 operational taxonomic units (OTUs) were identified for each sample. In this study, the major bacteria in the enterotypes identified from the fecal samples were *Bacteroides, Prevotella*, and Enterobacteriaceae, and their correlation with dietary habits was confirmed. A microbial interaction network in the gut was observed on the basis of the amount of short-chain fatty acids, pH value of the intestine, and composition of the bacterial community (enterotypes). Finally, a decision tree was derived to provide a predictive model for the three enterotypes. The accuracies of this model in training and independent testing sets were 97.2 and 84.0%, respectively.

**Conclusions:**

We used NGS technology to characterize the microbiota and constructed a predictive model. The most significant finding was that Enterobacteriaceae, the predominant subtype, could be a new subtype of enterotypes in the Asian population.

**Electronic supplementary material:**

The online version of this article (doi:10.1186/s12864-016-3261-6) contains supplementary material, which is available to authorized users.

## Background

Microorganisms inhabit various sites of the human body [1]. The largest number of microorganisms is found in the gut [1]. The gut microbiome is associated with human health [2]. For example, the gastrointestinal microbiome affects human physiological functions such as immune function and inflammation suppression, food decomposition and nutrient absorption, regulation of blood substrate via the nervous and/or endocrine system, and recovery rate from bacterial infection [3]. However, some of the underlying mechanisms remain unclear. Enterotypes of the human gut microbiome are not associated with gender, age, or body weight but are influenced by long-term dietary habits. Therefore, we aimed to identify the enterotypes of adults in Taiwan by next-generation sequencing (NGS).

Since the Human Microbiome Project (HMP) was launched by the National Institutes of Health in 2008, NGS has been widely used to study the human microbiome [4]. One of the benefits of NGS is that it is a culture-independent method that can be used to characterize microbial community profiles by sequencing of 16S ribosomal DNA (rDNA). In addition, hundreds to thousands of bacteria can be identified at a time on sequencing 16S rDNA by NGS. Thus, variations in bacteria among different samples can be determined by comparing their quantitative profiles [5].

In 2011, three enterotypes of the human gut microbiome were identified from 261 human fecal samples from European individuals. The major bacteria in these enterotypes were *Bacteroides*, *Prevotella*, and *Ruminococcus* [6]. This finding was subsequently validated by another approach using the same HMP dataset [7]. However, after identifying the long-term dietary habits in subjects, another study only observed *Bacteroides* and *Prevotella* enterotypes in their dataset and reported that *Ruminococcus* was an ambiguous enterotype [8]. The HMP dataset included gut microbiota that was rich in saturated fats and animal protein, whereas the latter study included microbiota from individuals with plant-based diets that were low in meat and high in carbohydrates [9]. In 2012, two other groups also reported that *Ruminococcus* could not be clearly classified in their datasets, and *Firmicutes* were identified as the dominant species in those studies [10, 11].

Based on the data from these previous studies, we were interested in determining if *Ruminococcus* is an enterotype in the gut microbiota of Taiwanese individuals. To this end, 181 human fecal samples from adults in Taiwan were collected, and the V4 regions of the 16S rDNA gene were sequenced through paired 150-cycle reads using the Illumina MiSeq system. A total of 17,675,898 sequencing reads were sequenced in 181 samples, and 215 operational taxonomic units (OTUs) were identified in each sample on average. The most abundant bacteria identified in the fecal samples were *Bacteroides, Prevotella*, and Enterobacteriaceae, and their correlation with dietary habits was confirmed. A decision tree model of these three enterotypes was constructed. The accuracies of this model in training and independent testing sets were 97.2 and 84.0%, respectively.

The most significant finding in our study was the identification of Enterobacteriaceae as one of the predominant subtypes in the gut microbiota. This species may be a new subtype of enterotypes in the Asian population.

## Results and discussion

### Sequencing data statistics

We conducted 17,675,898 sequencing reads on 181 stool samples. After filtering the sequences that did not fit the criteria, we further analyzed 16,474,959 sequencing reads. After taxonomy assignment, 9,133,183 sequencing reads were aligned to genes in the 16S rDNA database that had a sequence similarity of at least 97%; 215 OTUs for each sample were identified on average. Detailed information on the sequencing reads is listed in Additional file 1: Table S1.

### Enterotype identification in the fecal samples

**Fig. 1:**
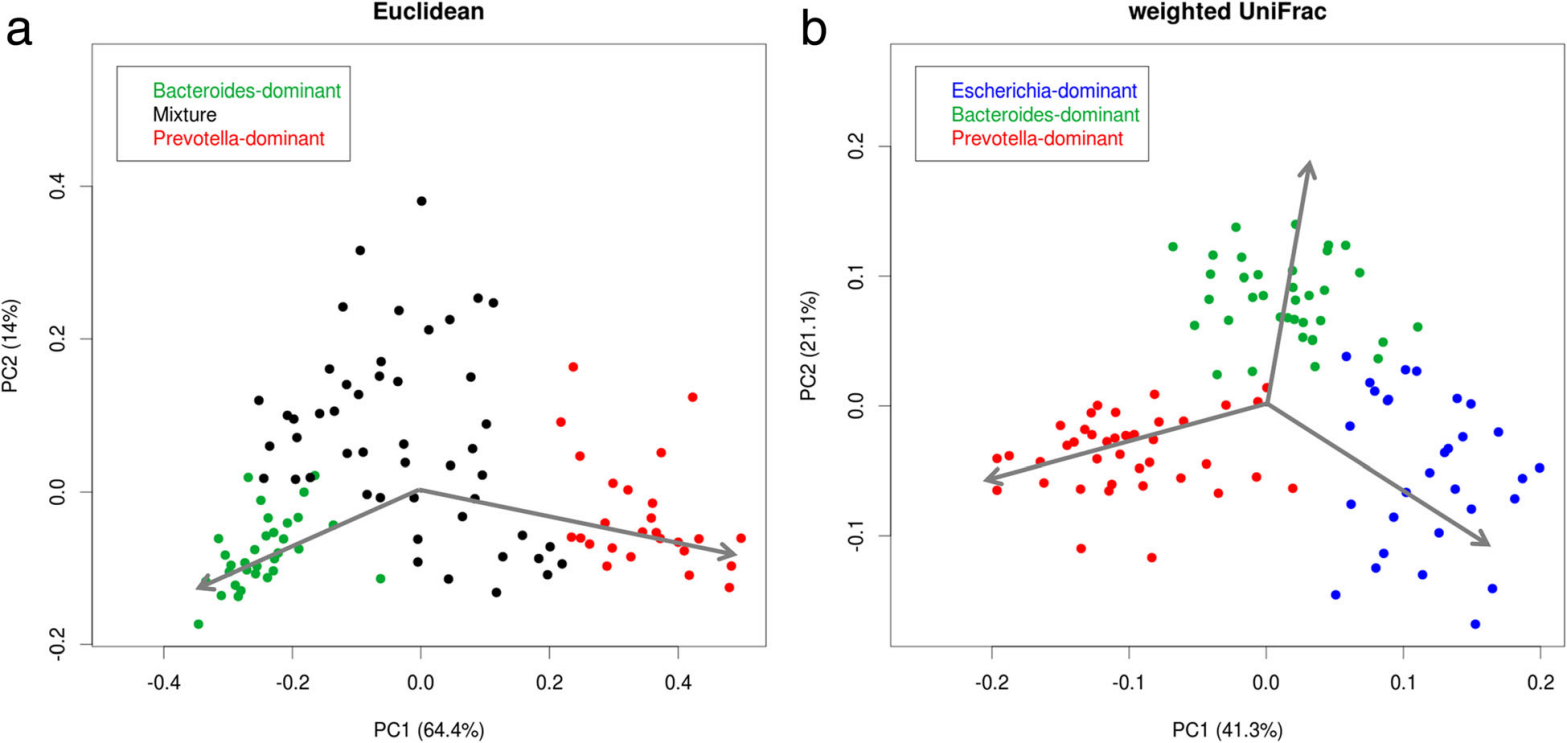
PCoA results based on different algorithms: **a** Euclidean and **b** Weighted UniFrac

**Fig. 2:**
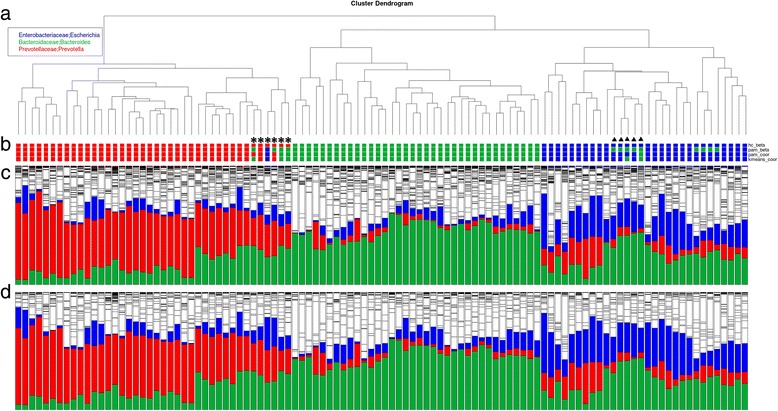
Hierarchical clustering results of the stool samples. **a** Phylogenetic tree of the stool samples; **b** enterotype of stool samples based on different algorithms (1^st^ row: hc_beta, 2^nd^ row: pam_beta, 3^rd^ row: pam_coor, 4^th^ row: kmeans_coor); **c** proportion of bacteria in the stool samples at the genus level (*blue*: *Escherichia*, *green*: *Bacteroides*, *red*: *Prevotella*); and **d** proportion of bacteria in the stool samples at the family level (*blue*: Enterobacteriaceae, *green*: Bacteroidaceae, *red*: Prevotellaceae)

Nine β-diversity matrices were used to identify the enterotypes in fecal samples via three clustering methods: hierarchical clustering (HC), partitioning around medoids (PAM), and k-means (Table 1). The optimal number of clusters was two using unweighted UniFrac distance. Principal coordinate analysis (PCoA) was also used to observe patterns in the stool samples. According to the Euclidean distance matrix, two clusters were shown in opposite areas (Fig. 1a, green and red dots), the major bacteria in which were *Bacteroides* and *Prevotella*. In contrast, the other samples (black dots in Fig. 1a) were scattered. Compared with the PCoA results of weighted UniFrac distance (Fig. 1b), the samples were grouped into three clusters, and the predominant bacterium in the third cluster was identified as *Escherichia*. Figure 2 shows the results of HC of the stool samples. The unsupervised classification method produced a dendrogram of the clustering results (Fig. 2a), and the results of four clustering algorithms of weighted UniFrac showed high concordance (Fig. 2b). Then three enterotypes were classified as containing *Escherichia* (enterotype 1), *Bacteroides* (enterotype 2), and *Prevotella* (enterotype 3) (Fig. 2c), and two small unconventional regions were found. One region was located in the *Prevotella*-predominant group (“star region” in Fig. 2b) and contained six samples. The abundance of *Prevotella* and *Bacteroides* showed a high positive correlation within the samples. In addition, nine samples, with a relatively high abundance of *Bacteroides* and low abundance of *Escherichia*, were classified into the *Bacteroides*-predominant group using the PAM method (“triangle region” in Fig. 2b). Moreover, the abundance of bacteria at the family level was similar to that at the genus level (Fig. 2d). Hence, the results of clustering may be improved on the basis of the family level.

**Table 1:** Summary of optimal cluster numbers

	HC^ac^	PAM^a^	PAM^b^	Kmeans^b^
Weighted UniFrac	3 (0.339)	3 (0.350)	3 (0.353)	3 (0.354)
Altgower	2 (0.280)	2 (0.161)	2 (0.305)	2 (0.309)
Bray	2 (0.302)	2 (0.297)	2 (0.309)	2 (0.309)
Jaccard	2 (0.221)	2 (0.216)	2 (0.225)	2 (0.225)
Kulczynski	2 (0.302)	2 (0.297)	2 (0.309)	2 (0.309)
Maximum	2 (0.459)	2 (0.451)	2 (0.464)	2 (0.468)
Pearson	8 (0.571)	2 (0.614)	2 (0.622)	2 (0.622)
Horn	2 (0.494)	2 (0.596)	2 (0.600)	2 (0.600)
Euclidean	2 (0.267)	2 (0.418)	2 (0.419)	2 (0.418)

The first number of each cell is the optimal cluster number and the second number is the Silhouette score. The optimal cluster number corresponding to the maximum score was picked from a limited series of cluster numbers (k ≤ 10)

^a^Beta matrix was applied as the input

^b^Coordinate (PC1–PC3) of each sample was applied as the input. Coordinate was generated by the classical multidimensional scaling method from the beta matrix

^c^HC, Hierarchical clustering

### Characteristics of the enterotypes

**Fig. 3:**
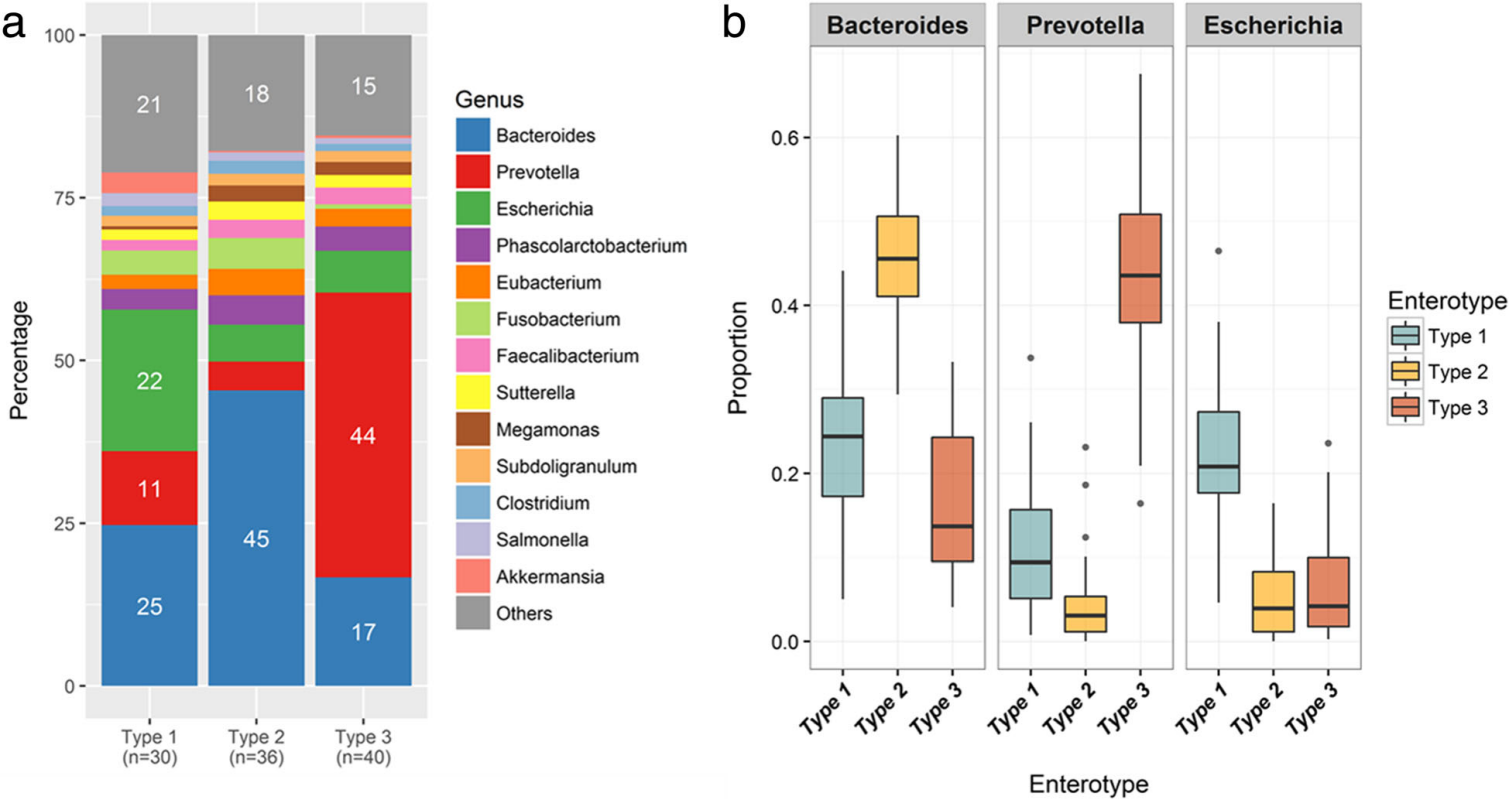
Bacterial community of three enterotypes: **a** bacterial proportion in the three enterotypes and **b** major bacteria in the three enterotypes

**Fig. 4:**
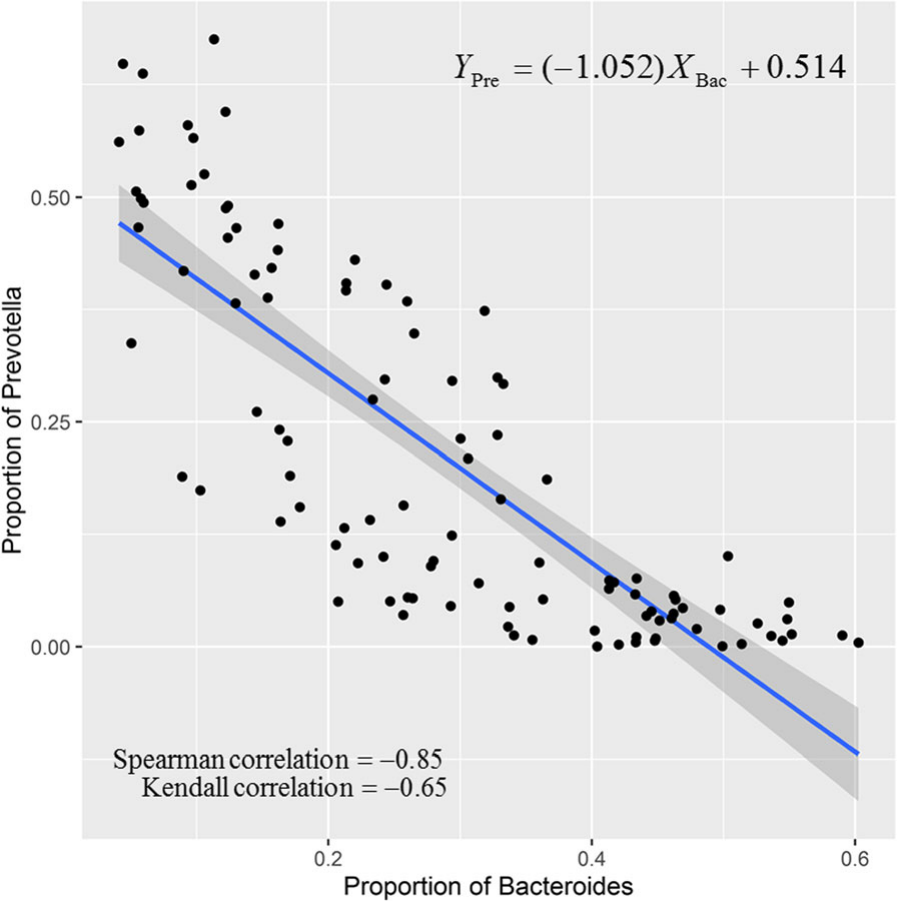
Linear regression results of abundance of *Bacteroides* and *Prevotealla* (Multiple R-squares: 0.658, adjusted R-squared: 0.654, and *p*-value < 2.2e-16)

The bar charts (Fig. 3a) illustrate the relative abundance of bacteria among the three enterotypes. In enterotypes 1 and 3, the major bacteria were *Bacteroides*, *Escherichia*, and *Prevotella*, which accounted for over half of the bacteria. In contrast, in enterotype 2, *Prevotella* were not the most abundant bacteria; the most abundant bacteria in this enterotype were *Bacteroides*, which accounted for almost half of the bacteria. In enterotypes 2, the abundance of *Bacteroides* was at least twofold higher than that of *Prevotella*. The relative abundance of *Escherichia* and *Bacteroides* in enterotype 1 was almost equal. Figure 3b shows the predominance of the three most abundant bacteria in the three enterotypes. The abundance of *Escherichia* was almost similar in both enterotypes 2 and 3, the abundance of *Bacteroides* in enterotype 1 was higher than that in enterotype 3, and the abundance of *Prevotella* in enterotype 1 was higher than that in enterotype 2. The proportion of *Bacteroides* was inversely correlated with that of *Prevotella* (*p* < 0.001, R = − 0.85) (Fig. 4). These results correspond to the bacterial abundance in enterotypes 2 and 3.

**Fig. 5:**
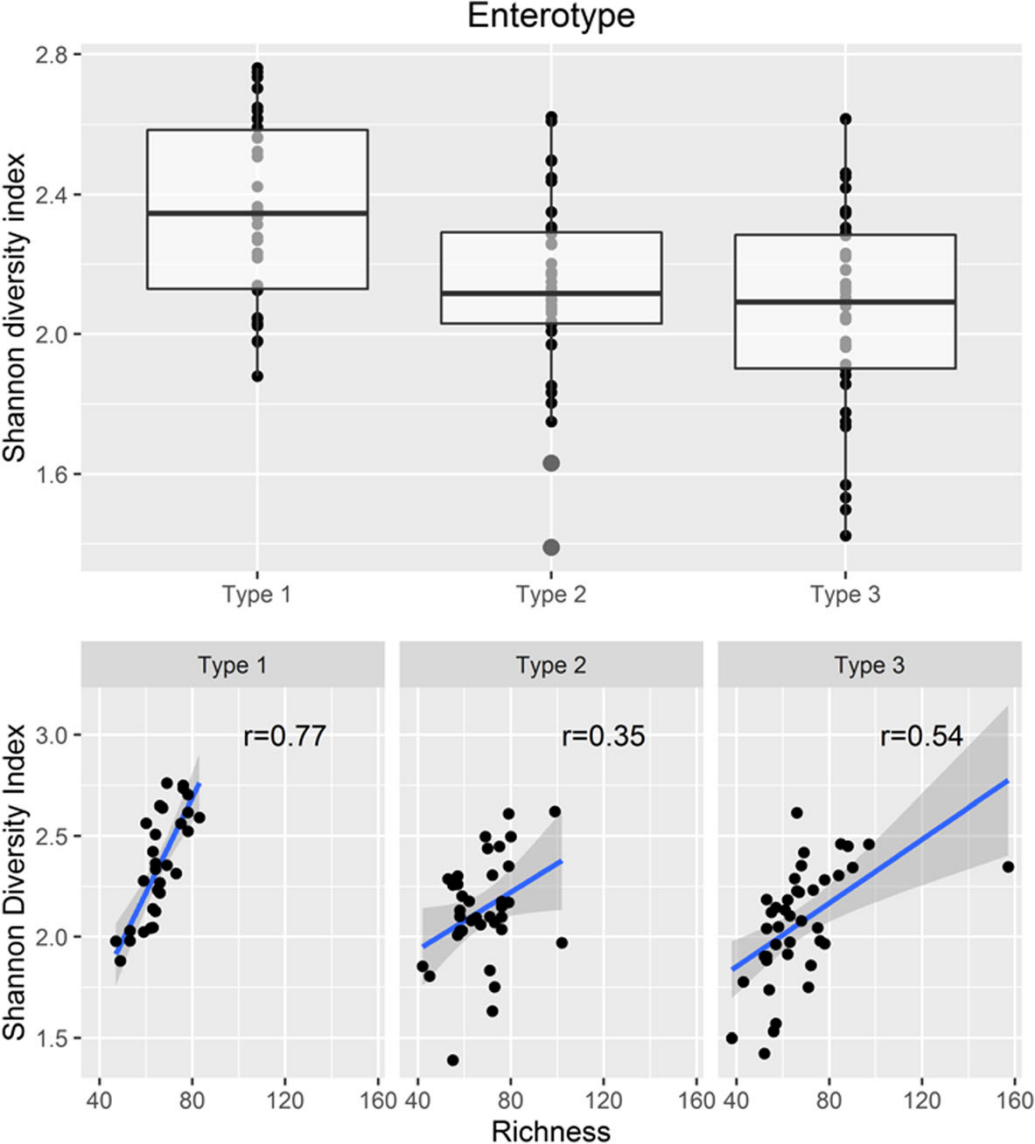
Distribution of the Shannon diversity index of three enterotypes and the correlation of richness and Shannon diversity index

**Fig. 6:**
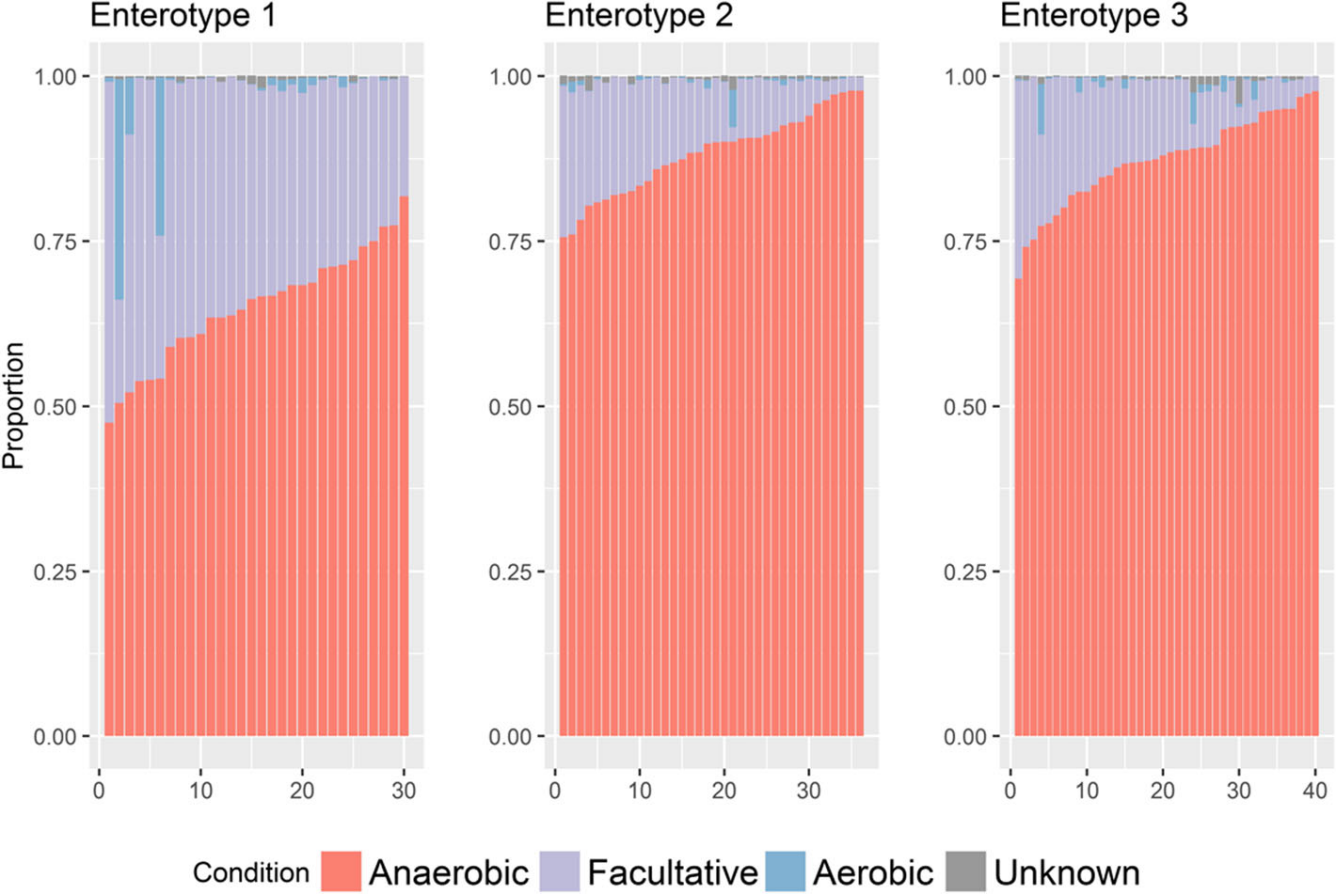
Composition of facultative, anaerobic, and aerobic bacteria of three enterotypes

The Shannon diversity index and composition of facultative, anaerobic, and aerobic bacteria in the three enterotypes are shown in Figs. 5 and 6, respectively. The *Escherichia*-predominant enterotype (enterotype 1) had a higher Shannon index than the other enterotypes (*p* < 0.001). Facultative and anaerobic bacteria showed an overwhelming majority (abundance of 98.3% on average) with a strong negative correlation (*p* < 0.0001, R = −0.94). It has been shown that anaerobic bacteria are the predominant bacteria at the endpoint of the gastrointestinal tract [12]. In our study, loss of anaerobicity was observed in the *Escherichia*-predominant enterotype, in which the abundance of facultative bacteria was significantly higher than that of the other enterotypes (*p* < 0.0001, fold change > 2.5) and significantly corresponded with a lower number of anaerobic bacteria (*p* < 0.0001). The facultative/anaerobic ratio (defined as the F/A ratio) was also significantly larger in enterotype 1 than in the other two enterotypes (*p* < 0.0001, fold change = 3.85).

### Enterotype phenotypes

The questionnaire given to the study subjects included questions about three major determinants regarding the samples. The first was the shape of feces, which was categorized by participants according to the Bristol stool scale. Scores of 1–3 represented “hard” stool, a score of four represented “mid,” and scores of 5–7 represented “watery” that had a high water content [13]. The second determinant was the frequency of excretion. At least one excretion every 2 days was designated “D1+,” excretion two to three times a week was designated “D05,” and excretion once a week or less referred to as “constipation.” The third variable was “protein type,” which referred to the major source of protein in daily diets: the non-red meat group included individuals who eat beans/vegetables, fish, and poultry and the red meat group included individuals who mostly eat livestock.

Table 2 demonstrates the association between enterotypes and several other factors such as gender, protein, shape, and stool frequency. Twice as many females as males were classified as enterotype 2 (T2 vs. T1 + T3, *p* = 0.02). In both enterotypes 1 and 3, the gender ratio was close to 1:1 (Table 2. Additional file 2: Figure S1a), which is similar to the results of previous studies [7, 10]. The number of individuals who consumed large quantities of red meat was twice the number of individuals who did not consume red meat consumers in enterotype 1 but half the number in enterotypes 2 and type 3 (T1 vs. T2 + T3, *p* = 0.0081) (Table 2. Additional file 2: Figure S1b). According to the results of the Bristol stool scale, feces shape showed a significantly higher correlation with enterotype 3 than water content (T1 + T2 vs. T3, *p* = 0.0038) (Table 2. Additional file 2: Figure S1c). Food digestion time (stool) in enterotype 1 was significantly higher than that in enterotypes 2 and 3 (T1 vs. T2 + T3, *p* = 0.0133) (Table 2). In addition, a higher digestion time was strongly associated with a high red meat diet (D1+ vs. D05, *p* < 0.0001, *χ* ^2^ = 12.92) (Additional file 2: Figure S1d).

**Table 2:** Association between enterotypes and various other factors from the questionnaire

	Enterotype			Association		
	Type 1 (*n* = 30)	Type 2 (*n* = 36)	Type 3 (*n* = 40)	Contrast	*p*-value	*χ* ^2^
Gender (global *p* = 0.047)				Type 1 vs Type 2	0.0336	4.52
male	17 (56.7%)	10 (27.2%)	19 (47.5%)	Type 1 vs Type 3	0.6046	0.27
female	13 (43.3%)	26 (72.2%)	21 (52.5%)	Type 2 vs Type 3	0.1258	2.34
				Type 2 vs (Type 1 + Type 3)^a^	0.0200	5.41
Protein (global *p* = 0.015)				Type 1 vs Type 2	0.0290	4.77
non-red-meat	8 (32.0%)	15 (68.2%)	18 (66.7%)	Type 1 vs Type 3	0.0264	4.93
red-meat	17 (68.0%)	7 (31.8%)	9 (33.3%)	Type 2 vs Type 3	1	0.01
				Type 1 vs. (Type 2 + Type 3)^a^	0.0081	7.00
Shape (global *p* = 0.014)				Type 1 vs Type 2	0.6133	0.98
Hard	10 (41.7%)	11 (55.0%)	3 (11.1%)	Type 1 vs Type 3	0.0356	6.67
Mid	8 (33.3%)	6 (30.0%)	11 (40.7%)	Type 2 vs Type 3	0.0320	11.51
Watery	6 (25.0%)	3 (15.0%)	13 (48.1%)	(Type 1 + Type 2) vs Type 3^a^	0.0038	11.15
Stool (global *p* = 0.064)				Type 1 vs Type 2	0.0384	6.52
D1+	17 (58.6%)	30 (83.3%)	30 (81.1%)	Type 1 vs Type 3	0.0711	6.29
D05	11 (37.9%)	4 (11.1%)	5 (13.5%)	Type 2 vs Type 3	0.9525	0.10
Constipation	1 (3.4%)	2 (5.6%)	2 (5.4%)	Type 1 vs (Type 2 + Type 3)^a^	0.0133	8.64

^a^Combining two types based on no significant difference between groups and closed trend

### Enterotype pathway enrichment analysis

Enterotype 1 shows higher pathway activity than enterotype 2 or enterotype 3 in some KEGG pathways (ko00902, ko00909, ko05168, ko05416, ko05145, ko05210, ko04115, ko04610) (Additional file 3: Table S2). Two metabolic pathways are related to terpenoid biosynthesis; three pathways are related to infections such as virus and parasite; two pathways are associated with cancer and p53 DNA repair system; ko04610 is related to innate immune system.

### Classification of enterotypes

**Fig. 7:**
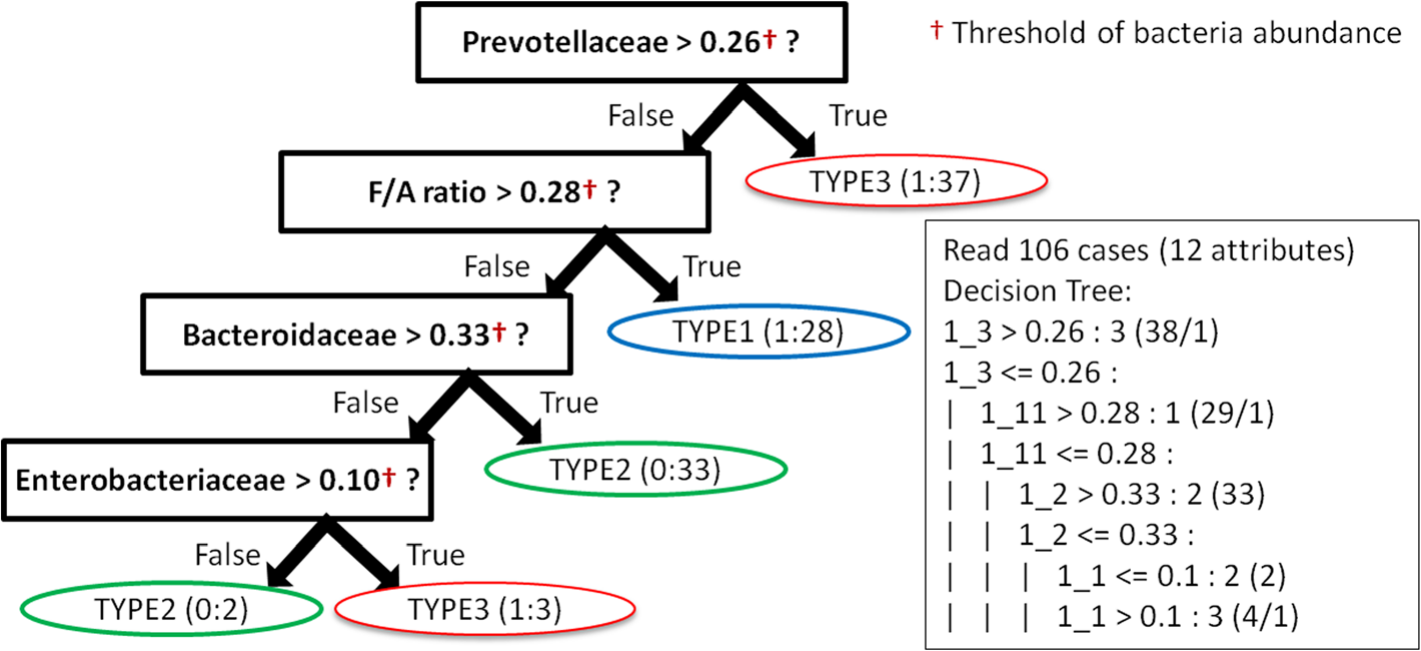
Decision tree model of the three enterotypes. This model provided five rules. Each rule could classify one of three enterotypes. For instance, if the bacterial abundance of Prevotellaceae was over 0.26 in one sample, the sample was considered enterotype 3 (1:37 means 37 samples were successfully classified and one sample failed)

To construct the decision tree model for classifying three enterotypes, 12 features were collected from 106 stool samples. These features include the Shannon index, F/A ratio, predominant genera and families, and enterotype-related phenotypes (gender, protein, shape, stool) (Additional file 4: Table S3). Figure 7 shows the result of the modeling. This decision tree interpreted five rules: 1) if the abundance of Prevotellaceae in a sample was greater than 0.26, the sample was considered enterotype 3; 2) if the abundance of Prevotellaceae in a sample was lesser than 0.26 and the F/A ratio was greater than 0.2, the sample was considered enterotype 1; 3) if the abundance of Prevotellaceae in a sample was lesser than 0.26, the abundance of Bacteroidaceae was greater than 0.33, and the F/A ratio was lesser than 0.28, the sample was considered enterotype 2; 4) if the abundance of Prevotellaceae and Bacteroidaceae in a sample was lesser than 0.26 and 0.33, respectively, the abundance of Enterobacteriaceae was greater than 0.10, and the F/A ratio was lesser than than 0.28, the sample was considered enterotype 3; and 5) if the abundance of Prevoteallaceae, Bacteroidaceae, and Enterobacteriaceae in a sample was lesser than 0.26, 0.33, and 0.10, respectively, and the F/A ratio was lesser than 0.28, the sample was considered enterotype 2. Rules 1–3 categorized 94.3% samples into three enterotypes. In total, 75 samples were used as independent testing sets to evaluate the performance of the decision tree model. Table 3 shows the performance of the training and testing sets, the accuracies of which were 97.2 and 84.0%, respectively.

**Table 3:** Performance of classification model in training sets and independent testing sets

		T1	T2	T3	Accuracy
Sensitivity	Train	93.3% (28/30)	97.2% (35/36)	100% (40/40)	97.2% (103/106)
	Test	93.8% (15/16)	79.3% (23/29)	83.3% (25/30)	84.0% (63/75)
Group-specific^a^ specificity	Train	98.7% (75/76)	100% (70/70)	97.0% (64/66)	
	Test	88.1% (52/59)	89.1% (41/46)	100% (45/45)	
Group-specific^a^ precision	Train	96.6% (28/29)	100% (35/35)	95.2% (40/42)	
	Test	68.2% (15/22)	82.1% (23/28)	100% (25/25)	

^a^Group-specific specificity, e.g., T1/non-T1

## Conclusions

This is the first study to identify enterotypes in stools from Taiwanese adults. Our findings revealed a new subtype of an enterotype predominant by family Enterobacteriaceae. The identification of this new subtype may have been due to the ethnic group, dietary habits, and locations studied. A decision tree model of enterotypes was constructed, and the accuracies of the training and independent test used to fit the model were 97.2 and 84%, respectively, which validated the model. Several associations between dietary habits and enterotype were identified in this study. Table 4 shows the predominant bacteria based on statistical hypothesis tests and several features. The microbial interaction network showed three bacteria (*Escherichia*, *Salmonella*, and *Klebsiella*), which belong to the same family (Enterobacteriaceae). In contrast to our findings, Enterobacteriaceae was found in a large number of patients with constipation from irritable bowel syndrome [14].

**Table 4:** Significant genus lists categorized by enterotype-related metadata

Features	Family	Genus	*p*. value	Mean separation
Enterotype	*Bacteroidaceae*	*Bacteroides*	<0.0001	T2 > T1 = T3
	*Prevotellaceae*	*Prevotella*	<0.0001	T3 > T1 > T2
	*Enterobacteriaceae* ^a^	*Escherichia*	<0.0001	T1 > T2 = T3
	*Enterobacteriaceae* ^a^	*Klebsiella*	0.0006	T1 > T2 = T3
	*Enterobacteriaceae* ^a^	*Salmonella*	0.0060	T1 > T3
	*Bifidobacteriaceae*	*Bifidobacterrium*	0.0394	T1 > T3
	*Pseudomonadaceae*	*Pseudomonas*	0.0198	T1 > T2
	*Verrucomicrobiaceae*	*Akkermansia* ^b^	0.0017	T1 > T2 = T3
Stool	*Veillonellaceae*	*Dialister*	0.0423	D1 + < D05
	*Verrucomicrobiaceae*	*Akkermansia* ^b^	0.0012^§^	D1 + < D05
Shape	*Porphyromonadaceae*	*Parabacteroides*	0.0106	Hard > Watery
	*Prevotellaceae*	*Prevotella*	0.0056	Hard > Watery
Protein	*Bifidobacteriaceae*	*Bifidobacterium*	0.0170	Red > non-red
	*Veillonellaceae*	*Megamonas*	0.0268	Red < non-red
	*Verrucomicrobiaceae*	*Akkermansia* ^b^	0.0042	Red > non-red

§*p* value was calculated by ANOVA (not significant in the Kolmogorov–Smirnov test)

^a^Genus within the same family

^b^Genus associated with multiple metadata

Enterotype-related phenotypes provide data for observing the gastrointestinal tract with the nature of continual flux [7], e.g., nutrient substrate, water context, or transition status. With regard to stool frequency, the amount of *Dialister* and *Akkermansia* in “D05” was higher than that in “D1 + .” According to previous studies, the amount of *Dialister* was higher in individuals with a high protein diet [15] and the amount of *Akkermansia* was higher in those with a fiber-free diet [16]. With regard to shape, the amounts of *Parabacteroides* and *Prevotella* in hard stool were higher than those in watery stool. Previous studies also showed that the amount of *Prevotella* was higher in ethnic groups that had a high fiber diet and lower in ethnic groups that adopted a Western diet [17]. With regard to protein type, the red meat group had abundance of *Bifidobacterium* and *Akkermansia* and the non-red meat group had abundance of *Megamonas*. Higher levels of lipid in the diet increased the amount of *Bifidobacterium* because it has the ability to digest lipids [18]. Enterotype 1 lacks of predominant bacteria such as *Prevotella* and *Bacteroide*, which may lead to a functional imbalance or a potential infectious risk via KEGG pathways (Additional file 3: Table S2).

Microflora, enterotype-related phenotypes, and short-chain fatty acids (SCFAs) were theoretically interwoven as a large association network [19]. Our study validates the connections among those factors (Additional file 5: Figure S2). SCFAs are byproducts of dietary fiber fermentation through microbiota, and they predominantly include acetic, propionic, and butyric acids. SCFAs can promote the growth of bacteria and can be absorbed by humans. Different types of SCFAs are sources of energy in different organs and are associated with intestinal diseases. Several factors control SCFA production in the gut, such as the amount and type of bacteria and the food retention time. The amounts of SCFAs affect pH of the intestine, for example, a higher concentration of SCFAs leads to lower pH. The pH value is associated with the composition of the bacterial community. The complex interaction network in the gut includes the amount of SCFAs, pH value of the intestine, and composition of the bacterial community.

Our results provide a predictive model for further analysis and new insights into enterotypes. An individual may change his/her enterotype by making dietary changes because the characteristics of enterotypes depend on an individual’s dietary habits. Although some researchers pointed that the gut microbiome should not category as ‘Enterotypes or Faecotypes’ since there is no clearly separation among clusters [20]. The classification may be blurred, yet the different features are still there. Thus, knowing one’s enterotype may allow doctors to outline the best diet for patients and to prescribe the most effective drugs.

## Methods

### Feces sample collection

The 181 human feces samples used in this population-based study were collected by Sigma-Transwab (Medical Wire). Feces were temporarily stored at 4 °C before DNA extraction. The exclusion criteria were age less than 10 years, a history of gastrointestinal tract surgery, and hospitalization or antibiotic treatment within the past 2 months. Of the resulting study cohort of 181 individuals, 106 provided complete information on the questionnaire and 75 omitted some information.

### DNA extraction

In this case study, fresh feces were obtained from participants, and DNA was directly extracted from stool samples using the QIAamp DNA Stool Mini Kit (Qiagen). A swab sample was vigorously vortexed and incubated at room temperature for 1 min. Then, the sample was transferred to a microcentrifuge tube containing 560 μl Buffer ASL, vortexed, and incubated at 37 °C for 30 min. Following this, the suspension was incubated at 95 °C for 15 min, vortexed, and centrifuged at 14,000 rpm for 1 min into pellet stool particles. Extraction was performed following the protocol of the QIAamp DNA Stool Mini Kit. The DNA was eluted with 50 μl Buffer AE and centrifuged at 14,000 rpm for 1 min, after which the DNA extract was stored at −20 °C until further analysis.

### Library construction and sequencing of the V4 region of the 16S ribosomal DNA

The PCR primers F515 (5′-GTGCCAGCMGCCGCGGTAA-3′) and R806 (5′-GGACTACHVGGGTWTCTAAT-3′) were designed to amplify the V4 region of the bacterial 16S ribosomal DNA as described previously [21]. PCR amplification was performed in a 50-μl reaction volume containing 25 μl 2X Taq Master Mix (Thermo Scientific), 0.2 μM of forward and reverse primer, and 20 ng DNA template. The reaction process increased the initial temperature to 95 °C for 5 min, followed by 30 cycles of 95 °C for 30 s, 54 °C for 1 min, and 72 °C for 1 min as well as a final extension of 72 °C for 5 min. Next, amplified products were checked by 2% agarose gel electrophoresis and ethidium bromide staining. Amplicons were purified using the AMPure XP beads (Agencourt) and quantified using the Qubit dsDNA HS Assay Kit (Thermo Fisher Scientific), all according to the respective manufacturers’ instructions. For V4 library preparation, Illumina adapters were attached to the amplicons using the Illumina TruSeq DNA Sample Preparation v2 Kit. Purified libraries were processed for cluster generation and sequencing using the MiSeq system.

### Filtering 16S rDNA sequencing data for quality

Sequencing reads from different samples were identified and separated according to specific barcodes at the 5’ end of the sequence (two mismatches allowed). The FASTX-Toolkit was employed to process the raw read data files. There were three steps used for sequence quality processing: (i) The command was “fastq_quality_filter –Q33 − q 20 − p 70.” “−q 20” meant that he minimum quality score to be maintained is 20. “−p 70” meant that the minimum percent of bases must have “−q” quality over or equal to 70%. (ii) The command was “fastq_quality_trimmer − t 20 − l 100 − Q33.” “−t 20” meant that bases with lower quality (<20) would be trimmed (checking from the end of the sequence). “−l 100” meant that the minimum acceptable length of sequence was 100 after trimming the sequence. (iii) Sequences were retained if both forward and reverse sequencing reads passed the first and second steps.

### Taxonomy assignment for bacteria 16S rDNA sequence

To generate taxonomy assignment, the collection of 16S rDNA sequences was retrieved from the SILVA ribosomal RNA sequence database (release 115) [22]. These sequences were extracted using V4 forward and reverse primers. Then, UCLUST was used to create representative sequence clusters over or equal to 97% similarity [23]. Bowtie2 was used to align sequencing reads against the clusters of the V4 sequence. A 97% similarity standard was applied to the V4 sequence clusters.

### Bacterial community analysis

After taxonomy assignment, an OTU table was generated. To normalize the sample size of all samples, a rarefaction process was applied to the OTU table. There are three steps in deciphering the enterotype of stool samples [24]. The first step is to calculate the distance matrix of β-diversity. R package “vegan” [25] and Python software “Pycogent” [26] were employed to calculate nine types of matrices, namely Alternative Gower, Bray Cutris, Jaccard, Kulczynski, Chebyshev, Pearson, Horn, Euclidean, and Weighted UniFrac. The second step is to use these matrices as the input data for three cluster algorithms: HC, k-means clustering, and PAM methods. For PAM, there were two types of inputs: one was the distance matrix of β-diversity and the other was the point information of XY axes that were transformed from the distance matrix. R package was also used to perform the clustering process. The third step is to evaluate the quality of clustering results. Silhouette score was calculated by R package “clusterSim” [27]. A higher score represents better quality of clustering results. To explore the association between bacterial community and factors related to individuals, which were extracted from the questionnaires, weighted α-diversity (Shannon index), chi-square test, and analysis of variance (ANOVA) were performed with R package. There were three criteria for identifying significant bacteria in the groups: the first was relative abundance > 1% in at least one group, the second was fold change in relative abundance between two groups ≥ log_2_(3) or ≤ log_2_(1/3). The third was *p* value ≤ 0.05. In order to construct a predictive model for classifying the three enterotypes of the stool samples, 181 stool samples were separated into two sets: the training set contained 106 samples, which were from individuals who provided complete information on the questionnaire, and the independent testing set contained 75 samples, which were from those who did not provide complete information. The decision tree, which was a rule-based machine learning method, was used to construct the predictive model for the three enterotypes. C4.5, which is a well-built decision tree package, was employed to perform this modeling process [28]. Tax4Fun was adopted to the pathway enrichment analysis with ANOVA [29].

## Additional files

Additional file 1: Table S1. Statistics showing analysis results of 181 stool samples. (XLSX 16 kb)
Additional file 2: Figure S1. Association between enterotypes and several other factors such as Sex, Protein, Shape and Stool. (TIF 11718 kb)
Additional file 3: Table S2 Pathway enrichment analysis. (PDF 235 kb)
Additional file 4: Table S3. List of selected twelve features. (XLSX 12 kb)
Additional file 5: Figure S2. Theoretical network of microflora, substrates of microbes, and transit time in human gut microsystem. (TIF 213 kb)
