# Supplementary figures and images for: Diversity and enterotype in gut bacterial community of adults in Taiwan
Source: BMC Genomics. 2017 Jan 25;18(Suppl 1):932. doi: 10.1186/s12864-016-3261-6 (PMC5310273; doi:10.1186/s12864-016-3261-6)

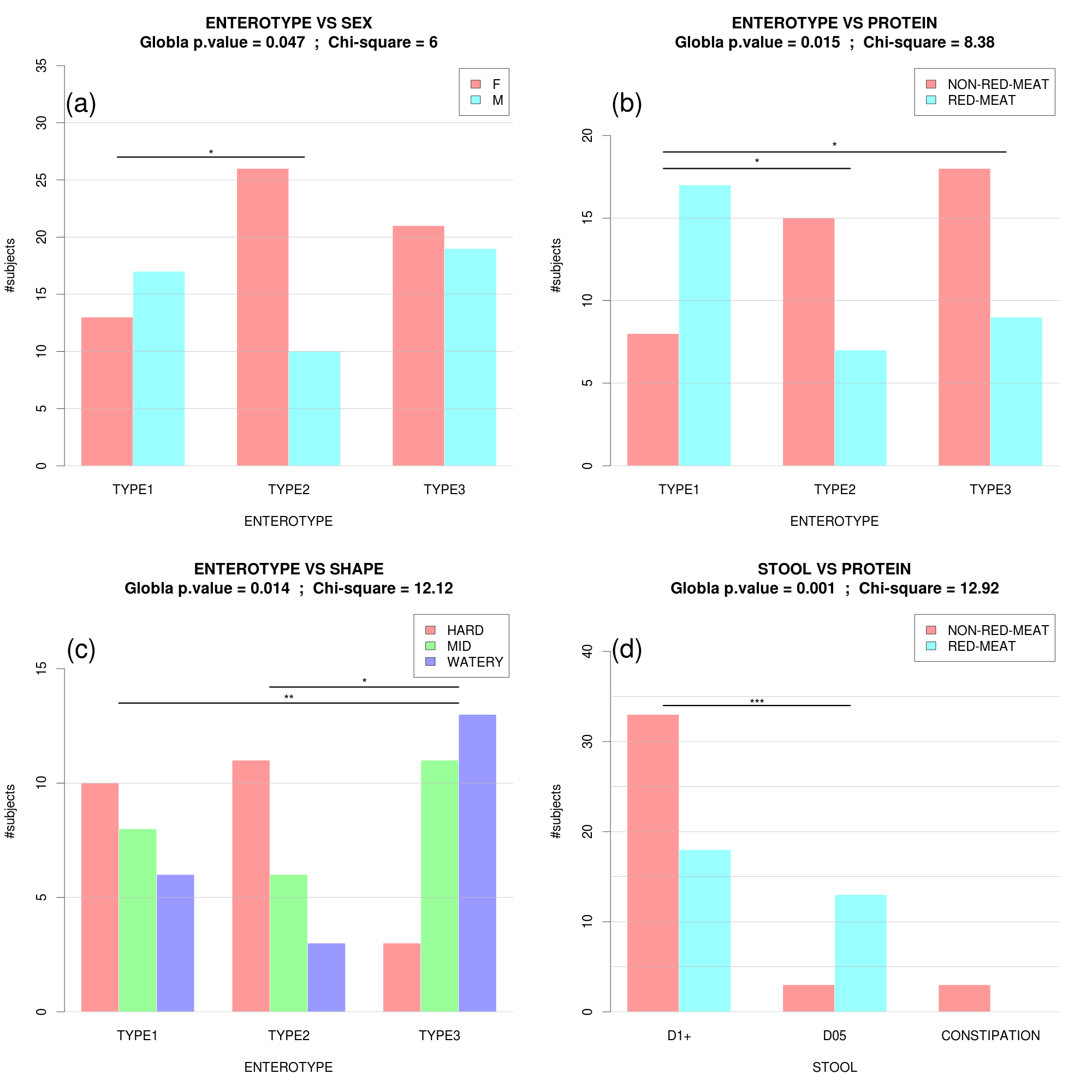

Supplement: Additional file 2: Figure S1. — Association between enterotypes and several other factors such as Sex, Protein, Shape and Stool. (TIF 11718 kb) [file 12864_2016_3261_MOESM2_ESM.tif]

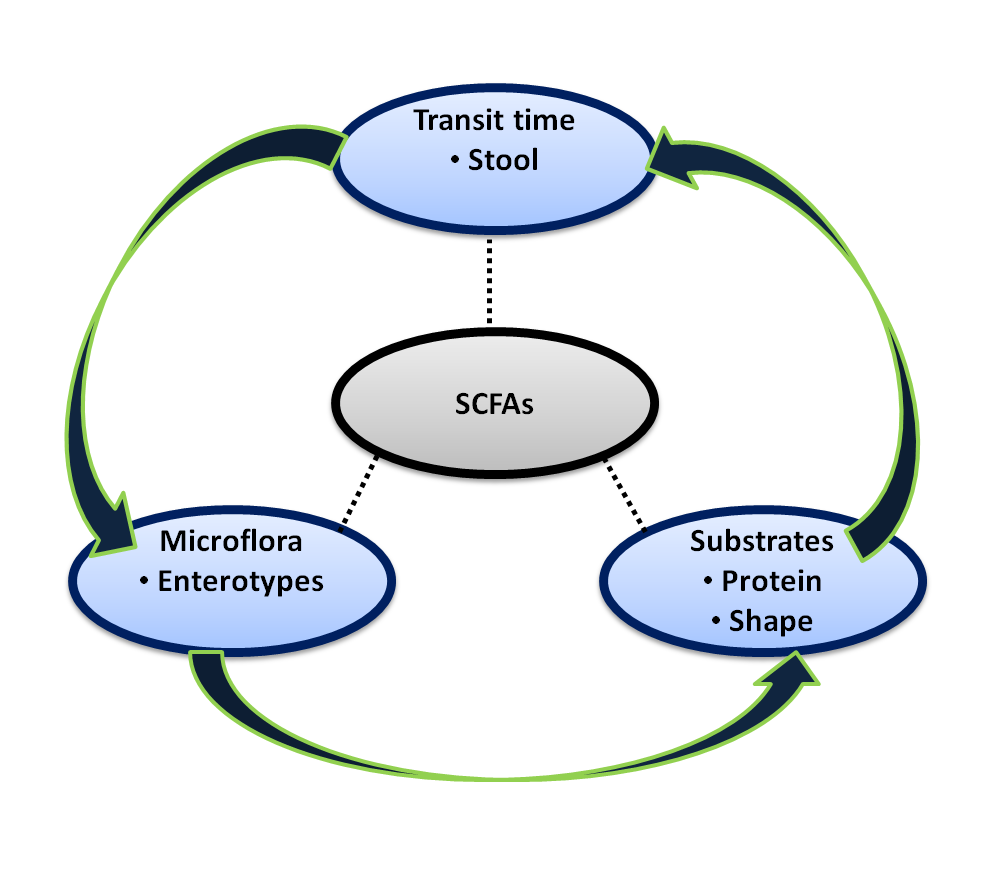

Supplement: Additional file 5: Figure S2. — Theoretical network of microflora, substrates of microbes, and transit time in human gut microsystem. (TIF 213 kb) [file 12864_2016_3261_MOESM5_ESM.tif]
