# Supplementary material for: Diversity and enterotype in gut bacterial community of adults in Taiwan
Source: BMC Genomics. 2017 Jan 25;18(Suppl 1):932. doi: 10.1186/s12864-016-3261-6 (PMC5310273; doi:10.1186/s12864-016-3261-6)
Supplement: Additional file 3: Table S2 — Pathway enrichment analysis. (PDF 235 kb) [file 12864_2016_3261_MOESM3_ESM.pdf]

Table S2 Pathway enrichment analysis

	Type 1[a]	Type 2	Type 3	p-value [b] T1 vs T2	T1 vs T3	T2 vs T3	Fold change [c] T2/T1	T3/T1	T3/T2	Effect [d]
ko00902 Monoterpenoid biosynthesis	1.7e-06 ± 3.9e-07	5.1e-07 ± 1.1e-07	6.8e-07 ± 1.3e-07	1.8e-03 *	5.6e-03 *	0.31	0.29	0.39	1.34	T1 > T2 = T3
ko00909 Sesquiterpenoid and triterpenoid biosynthesis	8.5e-05 ± 2.9e-05	7.8e-06 ± 1.4e-06	1.4e-05 ± 3.6e-06	4.2e-03 *	5.9e-03 *	0.11	0.09	0.17	1.82	T1 > T2 = T3
ko05168 Herpes simplex infection	1.6e-06 ± 4.5e-07	2.7e-07 ± 3.8e-08	3.9e-07 ± 7.6e-08	2.4e-03 *	4.1e-03 *	0.17	0.17	0.25	1.45	T1 > T2 = T3
ko05416 Viral myocarditis	1.5e-06 ± 4.6e-07	1.8e-07 ± 3.6e-08	3.1e-07 ± 6.4e-08	2.2e-03 *	3.8e-03 *	0.086	0.12	0.21	1.74	T1 > T2 = T3
ko05145 Toxoplasmosis	7.4e-06 ± 1.4e-06	3.2e-06 ± 4.7e-07	2e-06 ± 2.4e-07	4.3e-03 *	6.1e-05 *	0.017*	0.44	0.27	0.61	T1 > T2 > T3
ko05210 Colorectal cancer	1.5e-06 ± 4.6e-07	1.8e-07 ± 3.6e-08	3.1e-07 ± 6.4e-08	2.2e-03 *	3.8e-03 *	0.086	0.12	0.21	1.74	T1 > T2 = T3
ko04115; p53 signaling pathway	1.7e-06 ± 4.7e-07	2.9e-07 ± 7.2e-08	4.3e-07 ± 8.7e-08	1.8e-03 *	3.3e-03 *	0.21	0.17	0.25	1.51	T1 > T2 = T3
ko04610 Complement and coagulation cascades	1.6e-05 ± 2.4e-06	6.1e-06 ± 7.4e-07	4.9e-06 ± 6.5e-07	9.0e-05 *	4.7e-06 *	0.23	0.39	0.31	0.8	T1 > T2 = T3

a. Average pathway activity ± SEM

b. p-value: Evaluated by ANOVA; T1 means Type 1; T2 means Type 2; T3 means Type 3

c. Fold change (FC): T2/T1 means Type 2 is divided by Type 1, and etc.

d. Filtering criteria (1) p-value < 0.017 (2) The value of FC ≧ 3 or FC 1/3
